# Reliability of Self-Monitoring of Intraocular Pressure With iCare Home2 Rebound Tonometry

**DOI:** 10.1097/IJG.0000000000002560

**Published:** 2025-03-10

**Authors:** Dario Romano, Giovanni Montesano, Amir A. Aminoleslami, Benedetta Colizzi, Luca M. Rossetti

**Affiliations:** * Eye Clinic, ASST Santi Paolo e Carlo, San Paolo Hospital, University of Milan, Milan, Italy; † Department of Optometry and Visual Sciences, University of London; ‡ National Institute for Health Research (NIHR) Biomedical Research Centre at Moorfields Eye Hospital, NHS Foundation Trust and UCL Institute of Ophthalmology, London, UK

**Keywords:** glaucoma, intraocular pressure, self-monitoring, home-monitoring, rebound tonometry

## Abstract

**Précis:**

Using iCare Home2 (iCare, Finland) rebound tonometry, self-measurement of intraocular pressure has demonstrated good reliability and ease of use.

**Purpose:**

To investigate the reliability and repeatability of self-measured intraocular pressure (IOP) with rebound tonometry using iCare Home2.

**Patients and Methods:**

One hundred four patients out of 110 consecutive patients were recruited for this observational cross-sectional study. One randomly selected eye from each patient underwent 6 consecutive IOP measurements with Goldmann applanation tonometry (GAT), iCare IC200, and iCare Home2. Every eye was tested twice with each device, in random order, by an ophthalmologist for GAT and IC200, and by the patient itself for Home2. In addition, central corneal thickness (CCT) has been collected. The reliability of Home2 has been tested by calculating limits of agreement (LoA) between self-measured and physician-measured IOP, using the Bland-Altman analysis. The repeatability of each device has been tested by calculating the limits of repeatability (LoR) with the same method. Pearson correlation coefficient was used to determine the correlation between differences in IOP measurements and CCT.

**Results:**

The mean difference between GAT and iCare Home2 was −0.28±1.57 mmHg (*P*=0.070), 95%-LoA: (−3.36 to 2.79 mmHg﻿). The mean difference between IC200 and iCare Home2 was 0.92±1.48 mmHg﻿ (*P*<0.0001), 95%-LoA (−1.98 to 3.82 mmHg﻿). The mean difference between the first and second measurements with GAT, iCare IC200, and iCare Home2 measurements was 0.21±0.98 mmHg﻿ (*P*=0.03), −0.02±1.11 mmHg﻿ (*P*=0.88) and −0.23±1.04 mmHg﻿ (*P*=0.05).

**Conclusions:**

Self-measured IOP with rebound tonometry showed good reliability and repeatability when compared with physician-measured IOP with both standard GAT and rebound tonometry.

Elevated intraocular pressure (IOP) is the main risk factor for the development and progression of glaucomatous optic neuropathy.^1,2^ IOP is also the only modifiable risk factor to control the progression of the disease.^3^ IOP is subject to short and long-term fluctuations, and its variability have been found to be significantly higher in primary open angle and primary angle closure glaucoma eyes than in normal eyes.^4^ On the basis of this evidence, many authors have speculated about the impact of IOP fluctuation on visual field progression, but results are still controversial.^5–8^ One limitation in detecting short-term fluctuation is related to the fact that, in clinical practice, most ophthalmologist rely on sporadic measurements taken during office visits, and obtaining IOP measurements outside of normal office hours is uncommon. Moreover, it has been demonstrated how a single daily IOP measurement could lead to a chance higher than 75% of missing the peak of a diurnal curve.^9^ For this purpose, alternative tonometers should be considered to allow more frequent measurements. The Goldmann applanation tonometry (GAT) is still considered the gold standard to measure IOP,^10^ but it comes with several limitations that restrict its usage to clinical settings, such as the need for slit lamp, topical anesthesia, and trained personnel.^11^ Moreover, numerous sources of error and variability have been described, and its measurements could be influenced by both operator’s and patient’s issues.^12^

The iCare HOME (iCare, Finland) is a rebound tonometry derived from the standard iCare and specifically designed for self-IOP measurement.^13^ It has shown a good agreement with GAT and nowadays it is still the only tonometer available with this purpose.^14^ An updated version, the iCare HOME2, has been recently released with some improvements: it has a small display that shows the measured IOP, some sensors that automatically recognize the tested eye and the head position, and a colored LED light that guides the patient for correct positioning, thus providing a related quality score with each measurement.

Good reliability, when compared with GAT, has been found in a comparative retrospective study, but no data on self-measured IOP are yet available.^15^

The objective of this study is to assess the reliability and repeatability of self-measured IOP with the iCare HOME2, and to compare them with the IOP measured by the ophthalmologist with both iCare IC200 and GAT.

## MATERIAL AND METHODS

Participants were recruited among naive glaucoma patients and glaucoma suspects at ASST Santi Paolo e Carlo, Milan, Italy, following the acquisition of informed consent from all individuals. The protocol for this study was approved by the Institutional Review Board (Comitato Etico Milano Area 1, No. 0034559, July 31, 2023) and conducted in accordance with the principles outlined in the Declaration of Helsinki. Exclusion criteria included individuals with corneal abnormalities that could affect IOP measurements, as well as those with physical or mental impairments affecting their ability to use the tonometer. For each patient, only 1 eye randomly chosen was included in the study.

### Devices

The ICare rebound tonometer is a portable handheld device based on the principle of rebound tonometry.^16^ The ICare IC200 tonometer has an adjustable forehead support, an LCD display, and 4 buttons that can be used by the operator to take IOP measurements and to navigate through the history and settings menu. The device automatically takes 6 consecutive measurements, each of them is shown on the display, then excludes outliers, and calculates the average of the remaining 4 values.

On the basis of the same principle, the ICare Home2 is specifically designed for self-measurement. It differs from the IC200 model for the presence of double adjustable facial support and sensors that automatically recognize the tested eye and the head position and for a colored LED light that guides the patient for correct positioning. Both devices feature a color signal to evaluate the reliability of the result, marking unreliable measurements in yellow (significant variation among the 6 individual measurements) and reliable measurements in green. If the alignment is incorrect or the device is positioned too far or too close from the eye, the light turns red, and the measurement must be repeated.

Goldman applanation tonometry in the clinic served as a reference standard. The correct calibration of the tonometer (AT900, Haag-Streit, Köniz, Switzerland) was checked at the clinic every day, before taking the study measurements before the beginning of the study.

### Procedure

Intraocular pressure (IOP) measurements were obtained using the 3 instruments (GAT, ICare IC200, and ICare Home2) in a random order. IOP was measured with IC200 and GAT by an ophthalmologist masked to the IOP readings, in order to reduce bias. The patient performed self-tonometry with Home2, after appropriate training. All measurements were repeated twice to assess test-retest variability.

Enrolled patients underwent standardized training in the use of the Icare HOME2 to ensure patients’ proficiency and independence with the device. During the training period, patients were observed and instructed by a single experienced ophthalmologist. Once patients exhibited confidence in handling the device, they were asked to obtain 2 consecutive measurements independently. Only the measurements judged reliable were included for analysis. Any measurements resulted unreliable due to blinking or patient movements were discarded, and a new measurement was taken. If 2 consecutive iCare measurements failed, the patient was excluded from the study.

In all participants, central corneal thickness was measured using a specular microscope (Konan CellChek SL, Konan Medical Inc.). Additional data regarding the participants’ ocular history was obtained from clinical charts.

### Statistical Analysis

Statistical analysis was performed using R (R Foundation for Statistical Computing, ver. 4.3.3). Limits of agreement (LoA) and Limits of repeatability (LoR) were calculated using Bland-Altman analysis.^17^ Student-paired *t* test was used to compare the IOP measurements taken with different devices. Pearson correlation coefficient was used to determine the correlation between paired IOP measurements. The same statistical analysis was performed to determine the correlation between the difference among paired IOP values taken with different devices and CCT. As the iCare IC200 was the only device giving decimal IOP readings, all the analyzes were finally repeated, rounding those measurements to integers.

## RESULTS

A total of 110 patients were enrolled for this study. Four patients were excluded due to their inability to obtain at least 50% of reliable self IOP-measurements with iCare HOME2 and other 2 patients were excluded due to their excessive blinking, which made even the measurements taken by the medical staff unreliable. Finally, data from 1 random eye of 104 patients were considered for the analysis. The demographic features are shown in Table 1. The participating subjects had a mean age of 58.2±14.6 years and an average mean deviation on 24-2 visual field examination of −5.24±5.98 dB.

**TABLE 1:** Demographics and Baseline Characteristics of Study Participants

	n
No. patients	104
M/F	60/44 (*P*=0.117)
Mean age (y)	58.2±14.6 (38-77)
CCT (μm)	561±47 (445-710)
GAT (mmHg﻿)	17.23±9.00 (7-63)
iCare IC200 (mmHg﻿)	18.43±9.05 (7.9-62.3)
iCare Home2 (mmHg﻿)	17.51±9.19 (7-63)

The reported values are expressed as mean±SD, with their range shown in parentheses.

The mean measured intraocular pressure (IOP) was 17.23±9.00 mmHg﻿ with GAT (range: 7–63 mm Hg), 18.43±9.05 mmHg﻿ with iCare (range: 7.9–63.3 mmHg﻿), and 17.51±9.19 mmHg﻿ with iCare Home2 (range: 7–63 mmHg﻿).

**FIGURE 1:**
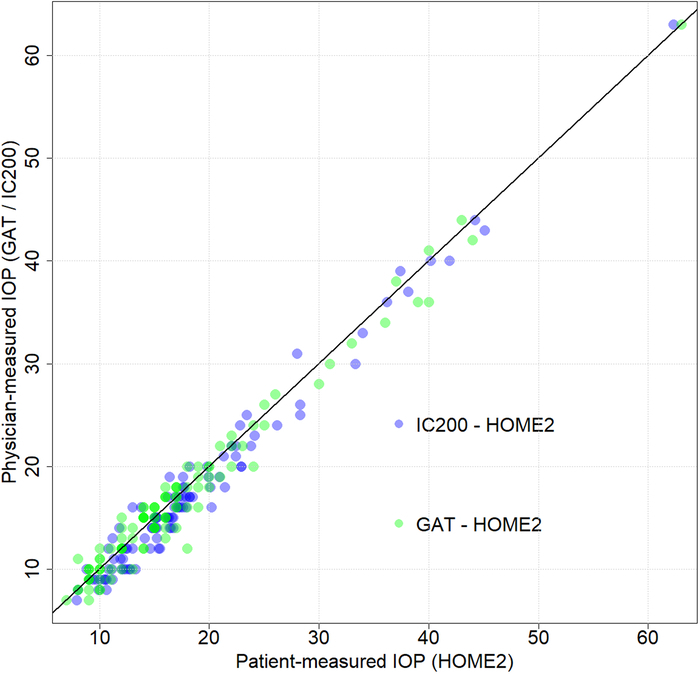
Scatter plot showing the correlation between paired patient and physician-measured IOP.

Paired IOP measurements between GAT and iCare, GAT and iCare Home2, iCare, and iCare Home2 showed a strong significant correlation (*r*=0.98; *P*<0.0001), (*r*=0.98; *P*<0.0001), and (*r*=0.98; *P*<0.0001) (Fig. 1).

**FIGURE 2:**
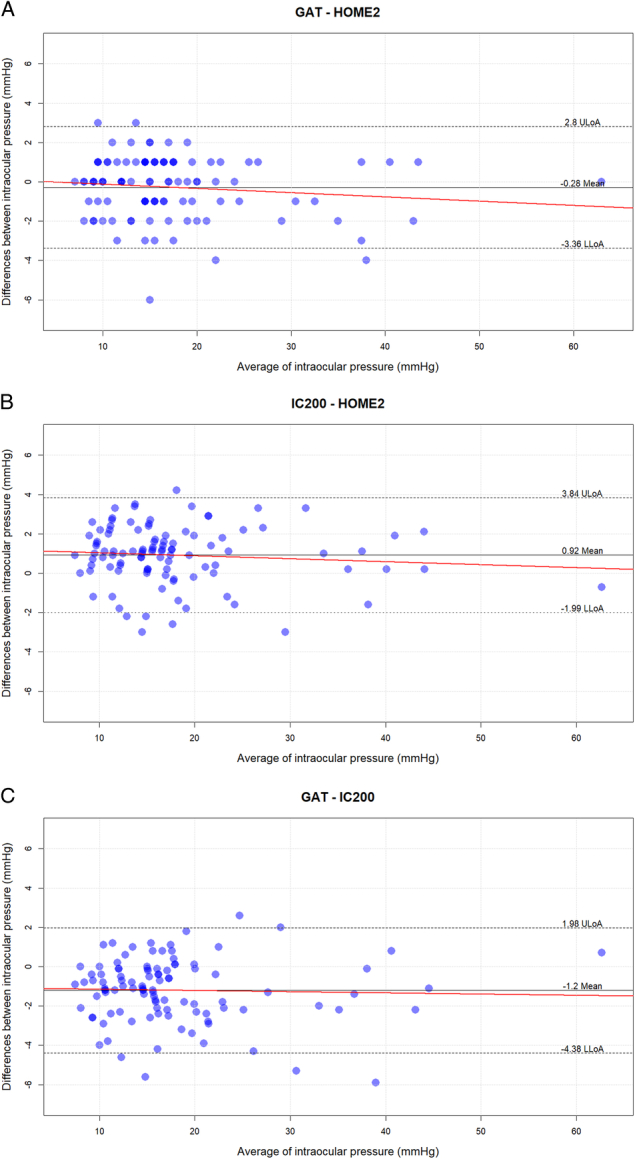
Bland-Altman plots illustrating the agreement between GAT and HOME2 (A), IC200 and HOME2 (B), GAT and IC200 (C). The area between the 2 dotted lines indicates the 95% limits of agreement on the paired measurements difference. The black solid line indicates the mean difference between paired IOP measurements with different devices. LLoA indicates lower limits of agreement; ULoA, upper limits of agreement. For each plot, a regression line is depicted in red.

The mean difference between GAT and iCare Home2 was −0.28±1.57 mmHg﻿ (*P*=0.070), 95%-LoA: (−3.36–2.79 mmHg﻿) and between iCare IC200 and iCare Home2 was 0.92±1.48 mmHg﻿ (*P*<0.0001), 95%-LoA (−1.99, 3.84 mmHg﻿). The mean difference between GAT and iCare IC200 was −1.20±1.62 mmHg﻿ (*P*<0.0001), 95%-limits of agreement (LoA): (−4.38, 1.98 mmHg﻿). Bland-Altman plots illustrating the agreement between the devices are shown in Figures 2A,B,C. A nonsignificant proportional bias was found for paired GAT and iCare measurements, meaning that with higher IOPs the IC200 yielded lower readings than GAT (*r*=0.056; *P*=0.57). In contrast, a weak negative correlation was found between GAT and Home2, with iCare giving proportionally higher measurements for higher GAT values (*r*=−0.037; *P*=0.79). A regression analysis showed a weak positive correlation among the differences between GAT and iCare measurements and the GAT readings (*r*=0.056; *P*=0.57). In contrast, we found a weak negative correlation among the differences between GAT and iCare Home2 measurements and the first GAT reading (*r*=−0.037; *P*=0.79). The same weak negative correlation was found among the differences between iCare and iCare Home2 measurements and the iCare readings (*r*=−0.17; *P*=0.79). With higher IOPs the iCare Home2 provided higher values compared with measurements taken by the operator, with both GAT and IC200, but the correlations were not statistically and clinically significant (0.006 mmHg﻿ for every GAT unit increase). In contrast, for every millimeter of mercury increase with GAT, the iCare IC200 gave an underestimation of 0.009 mmHg﻿.

**FIGURE 3:**
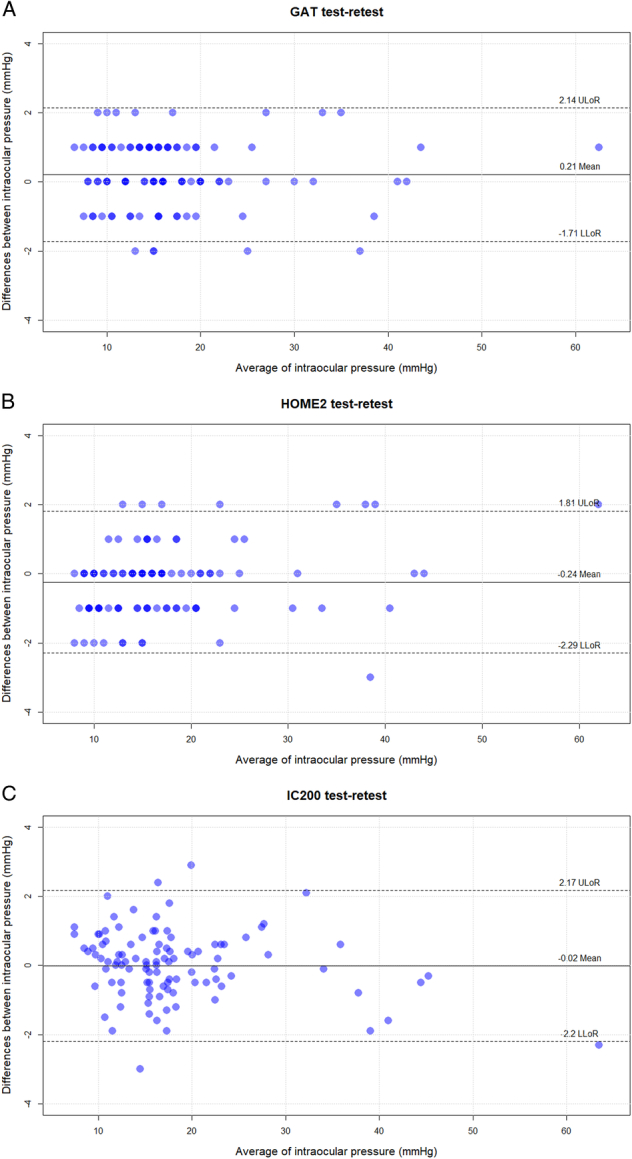
Bland-Altman plots of the test-retest variability for GAT (A), iCare Home2 (B), and iCare IC200 (C). The area between the 2 dotted lines indicates the 95% limits of agreement on the test-retest difference. The black solid line indicates the mean difference between test-retest measurements. LLoR indicates lower limits of repeatability; ULoR, upper limits of repeatability.

The mean difference between the first and second measurements with GAT, iCare, and iCare Home2 measurements was 0.21±0.98 mmHg﻿, 95%-limits of repeatability (LoR): (−1.71 to 2.13 mmHg﻿), −0.02±1.11 mmHg, 95%-LoR (−2.20 to 2.16 mmHg﻿), and −0.23±1.04 mmHg﻿, 95%-LoR (−2.27 to 1.81 mmHg﻿), respectively. The mean test-retest difference was significantly different from zero for GAT and iCare Home2 (*P*=0.03 and *P*=0.02, respectively), whereas the differences between the first and the second reading with iCare IC200 were not statistically significant (*P*=0.88), even if the IOPs were rounded to integer number (*P*=0.46). Bland-Altman plots of the test-retest variability for GAT, iCare Home2, and iCare IC200 are shown in Figures 3A,B,C.

**FIGURE 4:**
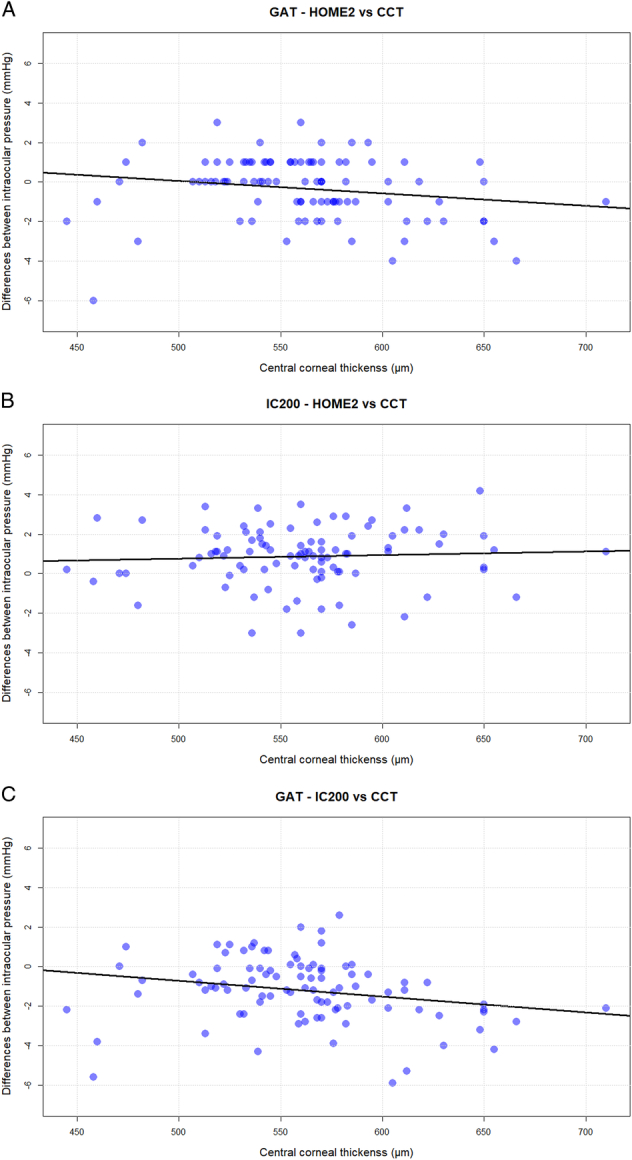
Scatter plots depicting the correlation among the CCT and the differences between GAT and iCare Home2 (A), iCare IC200 and iCare Home2 (B), and GAT and iCare IC200 (C). The black solid line indicates the tendency for thicker corneas to give proportionally higher IOP readings with rebound tonometry, compared with GAT.

Scatter plots depicting the correlation among the CCT and the differences between GAT and iCare Home2, iCare IC200 and iCare Home2, and GAT and iCare IC200 are shown in Figure 4. The plot demonstrates a bias, in which there is a negative correlation among both the difference between GAT and IC200 and between GAT and Home2 plotted against the CCT (*r*=−0.23, *P*=0.02 and *r*=−0.18; *P*=0.07), respectively. For CCT above 415 μm, the iCare readings were, on average, higher than Goldmann by 0.008 mmHg﻿ for every 1 μm increase in CCT. In the same way, when the cornea was thicker than 517 μm the iCare Home2 measurements were higher than Goldmann by 0.006 mmHg﻿ for every 1 μm increase in CCT. With corneas thinner than 415 and 517, rebound tonometry with IC200 and Home2, respectively, provided lower values than GAT.

## DISCUSSION

The results of this study have shown a good reliability and repeatability of rebound tonometry, when compared with applanation tonometry. To the best of our knowledge, this is the first study to evaluate the reliability and repeatability of self-measured IOP using the iCare HOME2 device. In addition, we compared data from both the iCare IC200 and HOME2, which utilize the same technology, to assess the specific impact of self-measurement on IOP readings. Both iCare IC200 and HOME2 tend to slightly overestimate IOP measurements when compared with GAT. The mean difference between iCare HOME2 measurements taken by the patients and those taken by the medical staff with GAT was −0.28±1.57 mmHg﻿ and despite the difference was found to be almost statistically significant (*P*=0.07), it is unlikely to be of clinical significance. Similarly, Kratz et al^15^ observed an even smaller mean difference when both GAT and HOME2 readings were obtained by an ophthalmologist, although with a wider 95%-LoA. Previous data on self-measured IOP with an earlier version of iCare Home showed good agreement with GAT, with a mean difference <1 mmHg﻿. In this study using the iCare Home2, we found 95%-LoA to be 50% narrower than previously reported, showing better reliability of the latest model.^14,18^ We also found good agreement between measurements taken by the same ophthalmologist with both GAT and iCare IC200 with a mean difference of −1.20±1.62 mmHg﻿. Even though the difference between these devices appeared to be statistically significant, it is close to 1 mmHg﻿ and would make little clinical impact in most clinical applications. Interestingly, we found a statistically significant difference between measurements obtained with IC200 and HOME2, despite both devices are sharing the same mechanisms. These differences may be attributed to the additional sensors and facial support in the HOME2 model, which require more precise centering to achieve reliable measurements. Despite the statistical significance, their clinical relevance remains debatable. The differences between GAT and rebound measurements showed a weak negative correlation with higher GAT values and vice versa. This indicates the tendency of rebound tonometers to overestimate higher IOPs and underestimate lower ones, both when used by the medical staff and by the patient itself. This is consistent to some findings previously described but, given the weak correlation, the clinical significance is questionable.^19,20^

All the 3 study devices provided excellent repeatability, as the mean difference between the first and second measurements with each of them was found to be not >0.23 mmHg﻿. Even though the test-retest differences were significantly larger for Home2 compared with GAT, the limits of agreement were overall very similar. The mean difference between the first and the second reading with iCare was the closest to zero (−0.02±1.11 mmHg, *P*=0.88), but the 95%-LoR were slightly wider than those of both GAT and Home2.

Both rebound and applanation tonometry appear to be affected by the CCT, however, we did not find a statistically significant effect. We found that the mean difference between GAT and IC200 and between GAT and Home2 becomes more negative with thicker corneas. These results were consistent with some findings previously published from the older iCare version,^21,22^ but in contrast to what Dabasia et al^18^ have found in a smaller cohort. Kratz and colleagues also investigated the effect of CCT on the difference between GAT and HOME2. Although, in their study, both applanation and rebound tonometry were performed by an ophthalmologist, they observed a similar CCT-related bias.^15^ However, CCT should be considered when evaluating IOP readings also with rebound tonometry, even though from our data, the impact of this correlation appears to be clinically relevant only for values above 600 μm. One of the participants was diagnosed with congenital aniridia and had a significantly thicker cornea (CCT 710 μm). As this condition is well known to be associated with increased corneal thickness,^23^ and given the absence of endothelial dysfunction or other corneal abnormalities, its data has been retained in the analysis. Moreover, correlations with other parameters were found to be similar to those observed in the other patients.

This technology would find particular use in frequent monitoring of IOP and in characterizing circadian variation.^6,9^ This would not be possible with sporadic IOP measurements obtained from in-clinic appointments. In contrast, measuring IOP at different hours in a standard clinic environment would require the patient to spend considerable time in the clinic and the presence of trained medical staff. At the moment, there are only 2 solutions for home monitoring of IOP, based on contact lens sensor (CLS) or rebound tonometry. The Triggerfish CLS (Sensimed, Lausanne, Switzerland) is the only wearable device CE-marked and FDA-approved, but it comes with a big limitation as it outputs values in units of millivolt equivalents, without a direct measurement of IOP. Both Mansouri et al^24^ and De Moraes et al^25^ tried to validate this method and to correlate 24-hours IOP fluctuation with VF progression, but the aforementioned limitation led to difficulty in the interpretation of the data and translation to clinical application. Moreover, the continuous wearing of a CLS could lead to blurred vision, foreign body sensation, ocular discomfort, risk of infections, and potentially reduction of topical medications absorption.^26^ In contrast, home monitoring with self-testing rebound tonometry could avoid all these limitations, but requires some manual dexterity and the ability to fixate the colored LED for correct alignment. Our cohort of glaucoma patients had a mean age of 58 years with mostly early to moderate glaucoma (average MD: −6.95±5.13 dB), and they were able to obtain reliable measurements in most of the cases. Only 4 patients have been excluded from the study due to their inability to obtain more than 50% of reliable readings. Three of them were older than 70 years and had advanced glaucoma (MD <12 dB), while a central visual field defect was present in 2 of the excluded patients. This should be kept in mind when employing this technology in practice, because not all patients might be good candidates for self-testing. Moreover, home rebound tonometry does not provide automated continuous measurements of IOP, thus it is less comfortable during night-time, as the patient needs to wake up to use the device.

In the last years, new developments in technology are creating the opportunity for the home monitoring of the visual field loss in glaucomatous patients, demonstrating a good agreement with standard automated perimetry.^27,28^ Such a technology could be ideally paired with home monitoring of IOP, providing a more complete picture for remote monitoring of glaucoma patients.

iCare Home2 has demonstrated good reliability and ease to use, making self-monitoring of IOP achievable and potentially useful in characterizing circadian variation and capturing IOP peaks. Further studies are needed to elucidate the impact of IOP fluctuation on visual field progression and to optimize the clinical application of this technology for glaucoma management.

## References

[1] Gordon MO Beiser JA Brandt JD . The ocular hypertension treatment study: baseline factors that predict the onset of primary open-angle glaucoma. Arch Ophthalmol. 2002;120:714–720. 12049575 10.1001/archopht.120.6.714

[2] Musch DC Gillespie BW Niziol LM . Intraocular pressure control and long-term visual field loss in the collaborative initial glaucoma treatment study. Ophthalmology. 2011;118:1766–1773. 21600658 10.1016/j.ophtha.2011.01.047 PMC3161134

[3] Spaeth GL . European Glaucoma Society Terminology and Guidelines for Glaucoma, 5th Edition. Br J Ophthalmol. 2021;105(suppl 1):1–169. 10.1136/bjophthalmol-2021-egsguidelines 34675001

[4] Sihota R Saxena R Gogoi M . A comparison of the circadian rhythm of intraocular pressure in primary phronic angle closure glaucoma, primary open angle glaucoma and normal eyes. Indian J Ophthalmol. 2005;53:243–247. 16333172 10.4103/0301-4738.18905

[5] Caprioli J Coleman AL . Intraocular pressure fluctuation. Ophthalmology. 2008;115:1123–1129.e3. 18082889 10.1016/j.ophtha.2007.10.031

[6] Asrani S Zeimer R Wilensky J . Large diurnal fluctuations in intraocular pressure are an independent risk factor in patients with glaucoma. J Glaucoma. 2000;9:134–142. 10782622 10.1097/00061198-200004000-00002

[7] Rabiolo A Montesano G Crabb DP . Relationship between Intraocular Pressure Fluctuation and Visual Field Progression Rates in the United Kingdom Glaucoma Treatment Study. Ophthalmology. 2024;131:902–913. 38354911 10.1016/j.ophtha.2024.02.008

[8] Barkana Y . Clinical utility of intraocular pressure monitoring outside of normal office hours in patients with glaucoma. Arch Ophthal. 2006;124:793. 16769832 10.1001/archopht.124.6.793

[9] Jonas JB Budde W Stroux A . Single intraocular pressure measurements and diurnal intraocular pressure profiles. Am J Ophthalmol. 2005;139:1136–1137. 15953461 10.1016/j.ajo.2004.12.012

[10] Goldmann H Schmidt TH . Applanation tonometry. Ophthalmologica. 1957;134:221–242. 13484216 10.1159/000303213

[11] Okafor KC Brandt JD . Measuring intraocular pressure. Curr Opin Ophthalmol. 2015;26:103–109. 25594767 10.1097/ICU.0000000000000129

[12] Whitacre MM Stein R . Sources of error with use of Goldmann-type tonometers. Surv Ophthalmol. 1993;38:1–30. 8235993 10.1016/0039-6257(93)90053-a

[13] Brusini P Salvetat ML Zeppieri M . How to Measure Intraocular Pressure: An Updated Review of Various Tonometers. J Clin Med. 2021;10:7–30. 10.3390/jcm10173860 PMC8456330 34501306

[14] Quérat L Chen E . iCare® Home vs Goldmann applanation tonometry: agreement of methods and comparison of inter-observer variation at a tertiary eye centre. Eur J Ophthalmol. 2023;33:312–318. 35505614 10.1177/11206721221099252 PMC9834317

[15] Kratz A Zbidat R Kishner R . Assessment of the iCare HOME2, a new intraocular pressure self-measurement tonometer. J Glaucoma. 2023;32:926–929. 37671544 10.1097/IJG.0000000000002298

[16] Cervino A . Rebound tonometry: new opportunities and limitations of non-invasive determination of intraocular pressure. B J Ophthalmol. 2006;90:1444–1446. 10.1136/bjo.2006.102970 PMC1857518 17114589

[17] Martin Bland J Altman Douglas G . Statistical methods for assessing agreement between two methods of clinical measurement. The Lancet. 1986;327:307–310. 2868172

[18] Dabasia PL Lawrenson JG Murdoch IE . Evaluation of a new rebound tonometer for self-measurement of intraocular pressure. Br J Ophthalmol. 2016;100:1139–1143. 26614630 10.1136/bjophthalmol-2015-307674

[19] Mudie LI LaBarre S Varadaraj V . The Icare HOME (TA022) Study. Ophthalmology. 2016;123:1675–1684. 27289178 10.1016/j.ophtha.2016.04.044

[20] Takagi D Sawada A Yamamoto T . Evaluation of a new rebound self-tonometer, Icare HOME: comparison with Goldmann Applanation Tonometer. J Glaucoma. 2017;26:613–618. 28369004 10.1097/IJG.0000000000000674

[21] Termühlen J Mihailovic N Alnawaiseh M . Accuracy of measurements with the iCare HOME rebound tonometer. J Glaucoma. 2016;25:533–538. 26859360 10.1097/IJG.0000000000000390

[22] Brown L Foulsham W Pronin S . The influence of corneal biomechanical properties on intraocular pressure measurements using a rebound self-tonometer. J Glaucoma. 2018;27:511–518. 29557828 10.1097/IJG.0000000000000948

[23] Whitson JT Liang C Godfrey DG . Central corneal thickness in patients with congenital aniridia. Eye Contact Lens. 2005;31:221–224. 16163015 10.1097/01.icl.0000152487.16012.40

[24] Mansouri K Weinreb RN Liu JHK . Efficacy of a contact lens sensor for monitoring 24-h intraocular pressure related patterns. PLoS One. 2015;10:e0125530. 25942434 10.1371/journal.pone.0125530 PMC4420265

[25] De Moraes CG Jasien JV Simon-Zoula S . Visual field change and 24-hour IOP-related profile with a contact lens sensor in treated glaucoma patients. Ophthalmology. 2016;123:744–753. 26854032 10.1016/j.ophtha.2015.11.020

[26] Garcia-Feijoo J Martinez-de-la-Casa JM Morales-Fernandez L . New technologies for measuring intraocular pressure. Prog Brain Res. 2015;221:67–79. 26518073 10.1016/bs.pbr.2015.06.003

[27] Harris PA Johnson CA Chen Y . Evaluation of the Melbourne rapid fields test procedure. Optom Vis Sci. 2022;99:372–382. 35383736 10.1097/OPX.0000000000001889

[28] Jones L Callaghan T Campbell P . Acceptability of a home-based visual field test (Eyecatcher) for glaucoma home monitoring: a qualitative study of patients' views and experiences. BMJ Open. 2021;11:e043130. 10.1136/bmjopen-2020-043130 PMC8030466 33820785

